# Effect of High-Intensity Interval Training Combined with Blood Flow Restriction at Different Phases on Abdominal Visceral Fat among Obese Adults: A Randomized Controlled Trial

**DOI:** 10.3390/ijerph191911936

**Published:** 2022-09-21

**Authors:** Shuoqi Li, Rong Guo, Tao Yu, Shiming Li, Tenghai Han, Wenbing Yu

**Affiliations:** 1 College of Physical Education, Yangzhou University, Yangzhou 225009, China; 2 School of Foreign Languages, Ludong University, Yantai 264025, China; 3 Department of Physical Education, Shandong Weihai Sports Training Center, Weihai 264400, China; 4 Department of Physical Education, Ocean University of China, Qingdao 266100, China; 5 Department of Physical Education, Weifang Medical University, Weifang 261053, China

**Keywords:** high-intensity interval training, blood flow restriction, visceral fat

## Abstract

Background: High-intensity interval training (HIIT) and blood flow restriction (BFR) represent a critical nonpharmacological strategy to reduce the excess deposition of visceral fat, as well as relevant complications, among obese populations. Applying BFR at diverse phases may have different effects. Therefore, the exercise program of this study combined HIIT with BFR, so as to explore the effect of BFR on abdominal visceral fat area and its mechanism in different periods of HIIT. The aim is to provide a more effective exercise prescription for obese people who want to reduce visceral fat quickly. Methods: This study was a randomized controlled trial involving 72 obese adults. One week before intervention, both regional and whole-body fat masses, abdominal subcutaneous and visceral fat areas, variables of blood metabolism, and VO_2max_ were recorded. Additionally, subjects with a matched fat percentage were randomized as a no-training control (C), HIIT (H), HIIT with BFR during interval (I), and HIIT with BFR during exercise (E) groups for 24 sessions within a 12-week period, using a cycle ergometer. During session one, this study recorded blood lactate, specific serum lipolytic hormones, rating of perceived exertion (RPE), and exercise heart rate (HR) and compared them among three groups. The baseline tests were repeated at 1 week after intervention. Results: There was no significant statistical difference in the indicators of each group at baseline (*p* > 0.05). The improvement of trunk fat mass and fat percentage of the I and E groups markedly increased relative to the H group (*p* < 0.05). Meanwhile, the I group had improved android fat mass and whole-body fat mass relative to group H (*p* < 0.05). Those exercise groups had markedly improved indices compared with the C group (*p* < 0.05). Additionally, the reduction in the I group had remarkably superior abdominal visceral fat areas (AVFA) to the H and E groups (*p* < 0.05). Immediately and 30 min following exercise, the E and I groups had remarkably increased growth hormone (GH) compared with the H group (*p* < 0.05). After exercise, the I group showed markedly increased epinephrine (EPI) compared with the H group (*p* < 0.05). The LA level in the I group evidently increased relative to the E group (*p* < 0.05), while that in the E group evidently increased compared with the H group (*p* < 0.05). Conclusion: Compared with HIIT alone, HIIT with BFR can better improve the body-fat level and glucose metabolism. HIIT with BFR in the interval phase better reduces the abdominal visceral-fat level than in the exercise phase, which may be due to the increase in lipolytic hormone level caused by the higher physiological load.

## 1. Introduction

Compared with peripheral obesity, visceral adiposity has been suggested to induce an increased risk of obesity-associated complications, such as cardiovascular disease (CVD) and type 2 diabetes mellitus (T2DM) [1,2,3], which is suggested to predict death among obese adult populations [4]. Eliminating excess visceral fat is related to attenuating metabolic syndrome, including reducing fasting plasma glucose (FPG) and expression of insulin resistance markers, which are more evident relative to symptoms induced by reduced subcutaneous fat [5,6].

Lifestyle modifications with high-intensity interval training (HIIT) represent the critical nonpharmacological strategy to reduce excess deposition of visceral fat, as well as relevant complications among obese populations [7,8,9]. A previous study showed that HIIT can further stimulate the secretion of catecholamines compared with moderate-intensity continuous training (MICT), which is more conducive to reducing visceral fat [8]. It is mostly related to lipolysis degree within visceral adipose tissues upon increased metabolic demands relative to subcutaneous fat [10], partly attributable to the higher activity of lipolytic hormones and lower activity of antilipolytic counterparts in the visceral adipocytes [11,12].

Blood flow restriction (BFR) training is a form of exercise that restricts the blood flow of the limbs, using a specific compression cuff [13]. BFR can promote the secretion of catecholamines by inducing distal muscle ischemia to increase metabolic pressure [14]. Recent studies confirmed that it can also be used in the field of fat reduction [15,16]. In addition, applying BFR during the interval phase of high-intensity resistance exercise can cause more blood lactic acid accumulation compared with that during the exercise phase [17]. On the one hand, the application of BFR during the interval phase may impair the recovery of blood flow and oxygen supply. On the other hand, when we apply BFR only during the exercise phase, high-intensity exercise itself may limit blood flow and reduce any beneficial effect of BFR. This indicates that applying BFR at diverse phases may have different effects.

Previous studies on BFR were mostly conducted on the basis of low-intensity aerobic exercise for exploring its impact on fat reduction [15]. However, due to this low intensity, it is unsuitable for obese adults who are eager to lose fat quickly. Therefore, the exercise program of this study combined HIIT with BFR, so as to explore the effect of BFR on abdominal visceral fat area and its mechanism in different periods of HIIT. It was hypothesized that the application of BFR in the interval phase may reduce abdominal visceral fat areas (AVFAs) better than in the exercise phase.

## 2. Methods

### 2.1. Participants

Seventy-two obese adults were recruited from Qingdao City, China, whose body fat percentage (fat%) was over 30% and who were willing to take part in the study. All enrolled participants had not undertaken regular aerobic training (less than once/week) for at least 1 year before initiating this work. Subjects conforming to the following criteria were excluded: (i) diabetes, hypertension, or angina pectoris; and (ii) exercise contraindications, such as heart or joint/bone diseases. All subjects were informed of the objective and procedure in the present work, provided informed consent for participation, and could quit the study freely in the case of any physical or psychological discomfort or tiredness. The Ethics Committee of the Ocean University of China (approval number: OUC-HM-2021016) approved our study protocols. This work was carried out in accordance with the Helsinki Declaration.

### 2.2. Study Design

**Figure 1:**
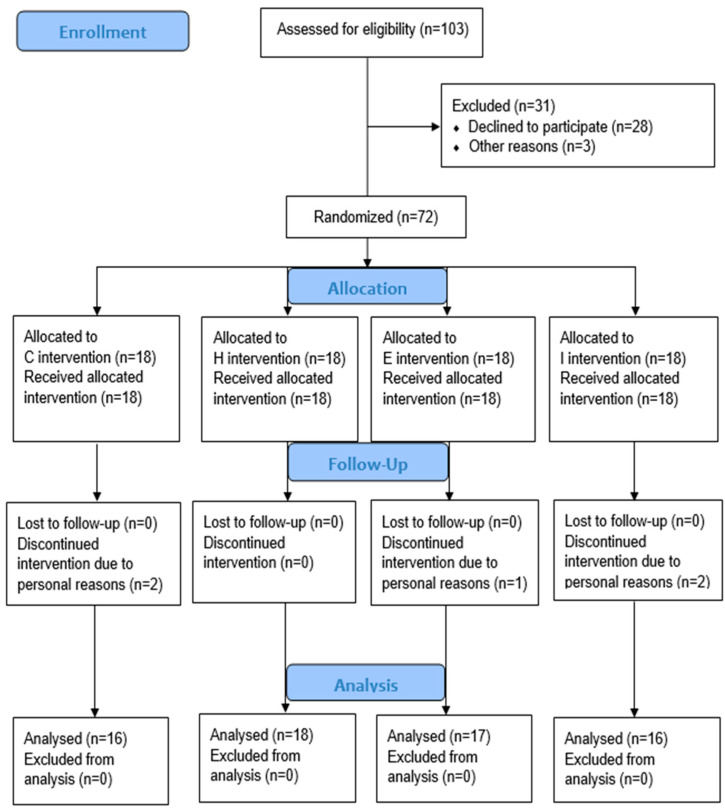
Flowchart of study design.

**Figure 2:**
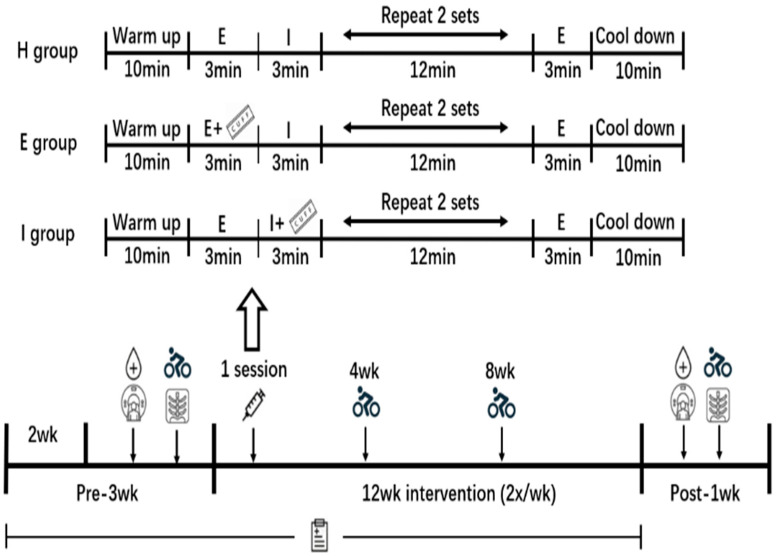
Experimental design and exercise program. Note: 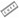 = phase of blood flow restriction; 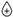 = fasting blood collection in the morning; 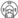 = dual-energy X-ray test; 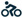 = maximum oxygen uptake test; 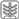 = computed tomography test; 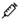 = blood collection before and after acute exercise.

Figure 1 and Figure 2 present the study-design flowchart. Briefly, daily food consumption, as well as physical activities, was recorded from 3 weeks before intervention to completion by all subjects to monitor habitual energy intake consumption. One week before intervention, both regional and whole-body fat masses, abdominal subcutaneous and visceral fat areas, variables of blood metabolism, and VO_2max_ were recorded. Additionally, subjects with matched fat% were randomized as no training control (C), HIIT (H), HIIT with BFR during interval (I), and HIIT with BFR during exercise (E) groups for 24 sessions within a 12-week period, using a cycle ergometer. The grouping information was given to the researchers in the form of sealed bags, and the participants did not know the actual content of the grouping. Five subjects quit the study, leaving 67 subjects (C group, *n* = 16; H group, *n* = 18; E group, *n* = 17; I group, *n* = 16). The intensity adopted by each group during exercise was 85% VO_2max_ intensity. At the end of weeks 4 and 8, VO_2max_ tests were repeated to adjust work rate on the basis of the predetermined training intensity. During session 1, blood lactate, specific serum lipolytic hormones, rating of perceived exertion (RPE), and exercise heart rate (HR) were recorded and compared among the three exercise groups. Baseline tests were repeated at 1 week after intervention.

### 2.3. Test Method for Calorie Expenditure and Intake

For all subjects, all daily energy intakes were recorded according to the 24 h dietary recall. A questionnaire was developed according to the Sports Nutrition Center of the National Research Institute of Sports Medicine (NRISM) guidelines in China to estimate caloric intake, for the sake of determining food and beverage consumption between midnight and midnight on the previous day. Dietary data were provided by each subject, including food type, preparation route, and portion-size guided by a dietician. In addition, the NRISM dietary and nutritional analysis system (version 3.1) was utilized for analyzing energy intake. In cases of violation while maintaining daily caloric intake, dietary advice was offered.

Daily physical activities, in addition to cycle ergometer training and sedentary activities, were evaluated according to the 24 h activity recall. The structured self-reported machine [8] was utilized to record physical activities between midnight and midnight on the previous day, which offered detailed information regarding physical-activity type and intensity, e.g., leisurely cycling and brisk walking. For each specific activity, the duration was measured for at least 5 min. Data regarding activities were transformed into energy-expenditure estimations, using the constructed MET codes from the Compendium of Physical Activity [18].

### 2.4. Body Composition Test

Blood metabolic variables and body fat mass were measured on the first day, whereas VO_2max_ and visceral fat area were determined on the following day. Tests before and after intervention were carried out by avoiding the menstrual period in female subjects and were of an identical order. When conducting the blood test and body composition test, each subject reported the results to the laboratory at 8:00 a.m. following at least 8 h of fasting, and strenuous exercises were avoided for a 48 h period. Thereafter, dual-energy X-ray absorptiometry (DEXA, Discovery Wi, Hologic Inc) was utilized to measure body-fat percentage, body mass, and fat masses in the trunk, whole body, and gynoid and android regions. The researcher was invited to adjust regional demarcations, following previous guidelines [19]. A computed tomography (CT) scanner (Somatom Definition Flash; Siemens) was utilized to assess cross-sectional abdominal subcutaneous (ASFA) and visceral fat areas, and the acquisition protocols were 150 mA and 120 kVp. In the test period, all subjects lay on their backs, with their arms naturally drooping. Images with a width of 5 mm at the umbilical level (about L4–L5 intervertebral space) were intercepted for evaluation. The volume calculation software embedded in CT scanner was adopted to evaluate ASFA and AVFA. In the scans, the voxel numbers within the whole dataset, with CT numbers from −190 to −30 HU, were drawn to present subcutaneous and visceral fat. ASFA and AVFA recorded by using the single-slice scan were closely related to (r ≥ 0.85) relevant umbilicus-level volumetric reconstructions [20]. The same technicians for CT and DEXA measurements were invited for tests before and after intervention, and they had no knowledge about the participants or intervention groups. The CV in observers was ≤3.5% and ≤5.6% for the measurement of fat variables by CT and DEXA, respectively.

### 2.5. Blood and Maximal-Oxygen-Uptake Tests

Blood was sampled from each subject in the seated position. Briefly, 5 mL of antecubital vein was harvested by means of venipuncture. An enzymatic assay (Jiancheng Biotech) was conducted for the immediate assessment of blood glucose (GLC), total cholesterol (TC), and triglyceride (TG). As for the remaining blood specimens, they were isolated for a 10 min period at 2000 r/min, followed by aliquoting and preservation at −80 °C to analyze serum insulin (INS) by adopting ELISA (RayBiotech). CV values within and between assays were ≤8.7% and ≤8.6% for variables.

The gradient cycling-exercise protocol initiating from 60 W (pedal frequency = 60 rpm) was adopted for determining VO_2max_. Power output was elevated by 40 and 20 W every 2 min for men and women until exhaustion. In our test period, HR and VO_2_ were determined by using an HR monitor (H12, Polar, Finland) and gas metabolism analyzer (Quark-PFT, COSMED, Rome, Italy), respectively. VO_2max_ represented the greatest mean values in 30 s.

### 2.6. Interventions

The duration of intervention was twice a week for 12 weeks. Exercise physiologists monitored each exercise to ensure safety. First, participants performed a 10 min warmup, with a 50 W load on the ergometer. After the warmup, each participant from different groups sprinted for 3 min with 85%VO_2max_ intensity; typically, the speed was kept at around 60 rpm, followed by resting on the ergometer for 3 min. Thereafter, the subjects were asked to sprint again, with the sprint and rest being repeated four times (Figure 2). During the training of the E and I groups, inflatable cuffs were placed at the inguinal folds of both legs to restrict blood flow. For the E group, BFR of 40% limb occlusive pressure (LOP) was applied in the process of exercise, whereas it was applied in the I group in the interval. Moreover, BFR was not applied in the C group throughout the process. Due to the high exercise intensity, participants could have adverse reactions, such as nausea, muscle cramps, or muscle soreness. Throughout the intervention, a researcher monitored the HR and RPE of participants. After exercise, activities were organized, and adequate emergency plans were prepared to ensure the safety of the participants.

### 2.7. Limb Occlusive Pressure

When measuring LOP, all the subjects wore a 7 cm–wide BFR cuff (The Occlusion Cuff, Belfast, Britain). The ultrasonic pocket Doppler (Edan, Northampton, UK) was utilized by researchers to measure leg arterial occlusive pressure (mmHg). Participants lay in bed with Doppler probes placed onto the tibial artery to capture auscultation pulses [21]. Thereafter, the cuff was placed within the thigh groin region, followed by slow inflation. The cuff pressure when the auscultation pulse was interrupted was defined as the LOP.

### 2.8. Statistical Analysis

G-Power software (Version 3.1.9.2, University of Trier, Trier, Germany) was utilized to determine the sample size by calculating power. The Shapiro–Wilk normality test revealed the normal distribution of variables. Differences in blood and body-fat variables, energy expenditure, and daily energy intake were assessed across diverse timepoints and different groups, using two-way ANOVA with repeated measures. In the case of a significant interaction, post hoc analyses for ANOVA were conducted to identify the simple main effects, using a Bonferroni test. A *p*-value < 0.05 denoted statistical significance. Data were presented as the mean ± standard deviation (SD).

## 3. Results

### 3.1. Participants

Table 1 displays the physical features of subjects from all the groups. No significant difference was identified in age, weight, height, or VO_2max_ among all the groups (*p* > 0.05). The sample size was estimated by using G*power by assuming a post hoc power (1 − β err prob) of 0.99.

### 3.2. Calorie Expenditure and Intake

Table 2 shows the calorie intake and expenditure in the 3 weeks before and 12 weeks during the intervention. Differences were of no significance between different groups prior to and after the intervention (*p* > 0.05).

### 3.3. Training Sessions

The compliance with interventional exercise of the C, H, E, and I groups was 98.1% ± 3.5%, 95.1% ± 3.6%, 97.5% ± 2.7%, and 98.2% ± 3.8%, respectively. Side-effects were not reported in each group during training or testing. Table 3 shows the means of exercise intensity, HR, RPE, and LOP for every 4 weeks of the intervention. There were no statistical differences between the baseline values of exercise intensity, HR, RPE, and LOP among diverse groups (*p* > 0.05). The exercise intensity of each group gradually increased during the intervention session, with a significant difference (*p* < 0.05). Other indicators of each group did not change significantly during the intervention process. The HR of the I group during exercise throughout the entire intervention process markedly increased compared with the H group. Other indicators were not significantly changed among groups.

### 3.4. Fat and Metabolic Variables

#### 3.4.1. Whole-Body and Regional Fat

Table 4 displays the body composition variables before and after intervention for the four groups, as well as the results of the repeated-measures ANOVA analysis. There were no significant differences in body-fat variables at baseline across the four groups (*p* > 0.05). Following intervention, all fat variables in each exercise group decreased significantly, except for the C group. The repeated-measures ANOVA results showed that the improvement in trunk fat mass and fat% of the I and E groups increased markedly relative to the H group (*p* < 0.05). Meanwhile, the I group had improved android fat mass and whole-body fat mass relative to group H (*p* < 0.05).

#### 3.4.2. Abdominal Visceral and Subcutaneous Fat

**Figure 3:**
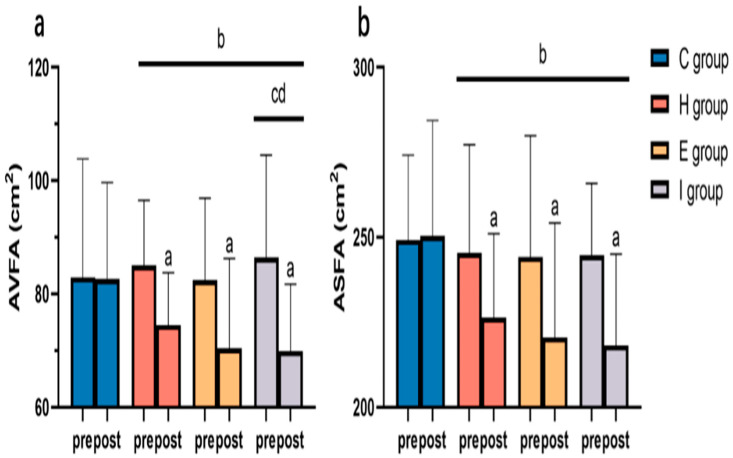
Changes in AVFA (**a**) and ASFA (**b**) before and after intervention. Note: ^a^ statistical difference relative to Pre; ^b^ statistical difference relative to C group; ^c^ statistical difference relative to H group; ^d^ statistical difference relative to E group.

Figure 3 shows the comparison of AVFA and ASFA in each group before and after the intervention. There was no statistical difference in the baseline value of each index among different groups (*p* > 0.05). After the 12-week exercise intervention, the indices of each exercise group decreased significantly compared with those before the intervention, while the C group did not show any significant difference (*p* < 0.05). As revealed by the repeated-measures ANOVA, exercise groups had markedly improved indices compared with the C group (*p* < 0.05). Additionally, the reduction in the I group had remarkably superior AVFA to the H and E groups (*p* < 0.05).

#### 3.4.3. Blood Glucose and Lipid Variables

**Figure 4:**
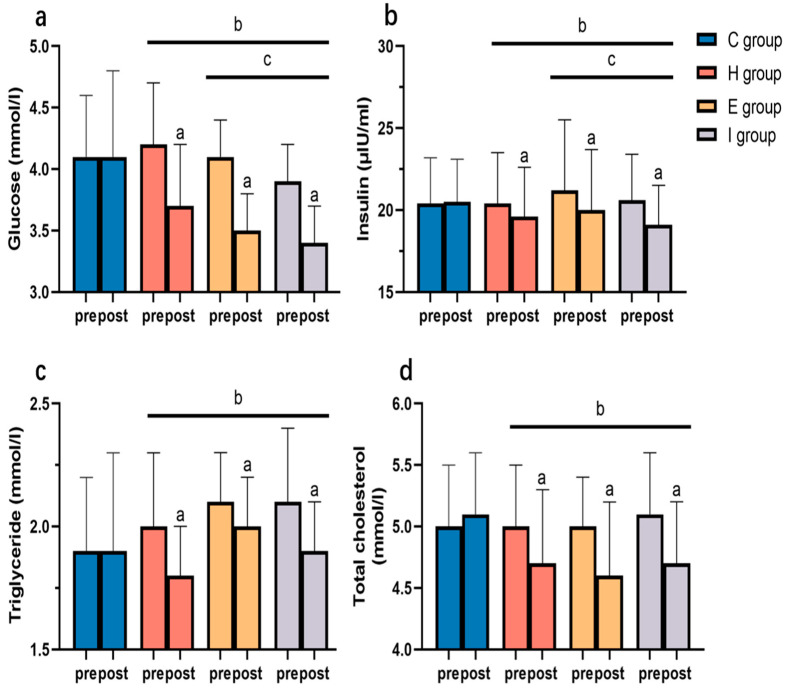
Changes in blood glucose (**a**), insulin (**b**), triglyceride (**c**), and total cholesterol (**d**) before and after intervention. Note: ^a^ statistical difference relative to Pre; ^b^ statistical difference relative to C group; ^c^ statistical difference relative to H group.

Figure 4 shows the comparison of blood glucose and lipid changes in each group in the morning in a quiet status before and after intervention. The differences in baseline index values were not significant among different groups (*p* > 0.05). After the 12-week exercise intervention, the indices of each exercise group decreased significantly compared with those before intervention (*p* < 0.05), while the C group did not exhibit any evident alteration (*p* > 0.05). As revealed by the results of repeated-measures ANOVA, each exercise group showed markedly superior improvements in all indices to those of the C group (*p* < 0.05). The improvements in blood glucose and insulin in the E and I groups were significantly better than those in the H group (*p* < 0.05).

#### 3.4.4. Lipolytic Hormones and Blood Lactate

**Figure 5:**
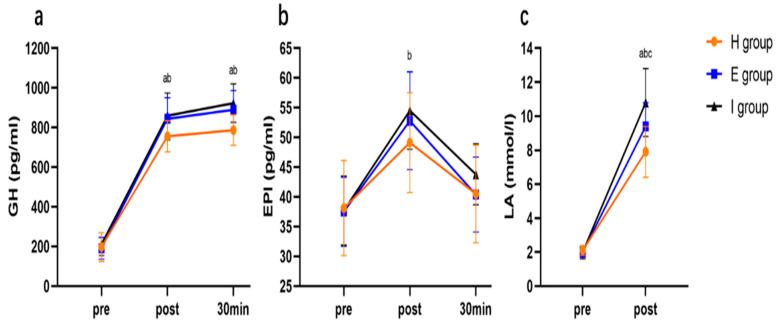
Changes in GH (**a**)**,** EPI (**b**), and LA (**c**) after exercise. Note: ^a^ statistical difference in E group compared with H group; ^b^ statistical difference in I group compared with H group; ^c^ statistical difference in I group compared with E group. GH = growth hormone; EPI = epinephrine; LA = lactic acid.

Figure 5 displays the serum growth hormone (GH), epinephrine (EPI), and LA levels of each exercise group prior to, immediately after, and 30 min following intervention in the initial session. The indices of each group before exercise did not exhibit any significant differences (*p* > 0.05). After exercise, GH, EPI, and LA increased significantly in all groups (*p* < 0.05). After 30 min of exercise, GH maintained its previous level, and EPI returned to the level before exercise (*p* < 0.05). Immediately and 30 min following exercise, the E and I groups had remarkably increased GH compared with the H group (*p* < 0.05). After exercise, the I group showed a markedly increased EPI compared with the H group (*p* < 0.05). After exercise, the LA level in the I group evidently increased relative to the E group (*p* < 0.05), while that in the E group evidently increased compared with the H group (*p <* 0.05).

## 4. Discussion

This study compared the fat reduction induced by exercise training during a 12-week HIIT program combined with BFR in different phases. The training intensity was 85% VO_2max_, and the total duration of each session was 36 min. According to our results, compared with the H and E groups, the I group exhibited greater visceral-fat loss after exercise. In addition, HIIT combined with BFR was more conducive to improving blood glucose metabolism and reducing body fat mass than HIIT alone. In general, among the three training programs with the same exercise duration, HIIT with BFR in the interval phase was the most effective strategy to combat central obesity.

Chen et al. [15] carried out an aerobic exercise program at 40% VO_2max_ among the population with obesity for a 12-week period. For the obese population showing reduced VO_2max_, BMI and fat% were markedly reduced. On the basis of the above results, cycling exercise with BFR represented the superior option for the obese population to aerobic exercise at approximately 40% VO_2max_ without BFR. This conformed to the results of this study, but it did not explore the effect of BFR training on visceral fat. Compared with subcutaneous fat, visceral fat exerts increased damage to health [1]. Kargaran’s study compared the efficacy of 8 weeks (three times per week) of dual-task training in the presence/absence of BFR on visceral fat mass in elderly women. The exercise intensity was 45% heart-rate-reserve intensity, and the BFR pressure was 50% LOP. According to their results, the BFR group more effectively improved visceral fat mass than the non-BFR group. This provides strong evidence that BFR promotes a reduction in visceral fat mass.

It is speculated that the main cause of this improvement in visceral fat may be ascribed to the excessive accumulation of lactic acid caused by the large physiological load during exercise. On the one hand, applying BFR in the interval phase possibly compromises oxygen supply and blood flow restoration [22], thus reducing ATP resynthesis [23]. Consistently, according to research applying high-load resistance exercise in the presence of BFR, the phosphocreatine level decreases, while the intramuscular inorganic phosphate level increases in the presence of BFR in the rest intervals in comparison with the protocol without BFR [17]. Furthermore, for BFR alone, high-intensity contraction may restrict blood flow, thereby reducing BFR’s benefits in the exercise interval. Additionally, blood flow can be restored by removing BFR interval, which allows for muscle-substrate recovery in the rest interval [24]. Therefore, relative to applying BFR in the exercise phase of HIIT, the application of BFR in the interval phase of HIIT further increases metabolic stress, while also maintaining high mechanical stress during the exercise process.

The increase in lactate level after exercise is closely related to the factors promoting fat reduction, including the lipolytic hormone and excess post-exercise oxygen consumption (EPOC) [25]. Firstly, the greater elevation of energy expenditure during exercise and EPOC may be factors responsible for the benefits of HIIT compared with BFR for fat loss [26]. The increase in energy expenditure during exercise and EPOC is also closely related to the level of blood lactic acid [27,28]. The results of this study showed that the blood lactic acid level of the I group after exercise was markedly elevated compared with the remaining groups. The research by Silva et al. [27] also supported the above hypothesis, showing that, compared with the non-BFR group, aerobic exercise combined with BFR effectively improved energy expenditure during exercise and EPOC. EPOC assists in restoring myoglobin and hemoglobin O_2_ levels, maintaining ventilation and heart rate after exercise, and returning to the creatine phosphate and intramuscular adenosine triphosphate levels adopted in the exercise process [29]. Therefore, intramuscular creatine phosphate content possibly decreases after exercise intervention, thus inducing the enhanced EPOC response in comparison with aerobic exercise in the presence of BFR, since aerobic exercise with BFR mainly depends on nonoxidative metabolism. Additionally, it is possibly associated with the increase in catecholamine release and accumulation of anaerobic metabolites during exercise. EPI is markedly related to EPOC duration and magnitude, which can increase recovery VO_2_ [30]. As revealed by Sedlock et al. [31], EPI elevated EPOC through promoting hepatic glucose release, as well as triglyceride/fatty acid cycling. In addition, EPOC might be decreased via a reduction in sympathetic nervous system activity. BLA is also strongly related to EPOC, especially the fast EPOC component. The metabolism of blood lactic acid after exercise is an important part of EPOC. As revealed by Aguiar et al. [25], EPOC might be associated with alterations of BLA in the initial recovery process following exercise. According to Frey et al. [32] BLA is significantly related to EPOC (r = 0.90). In conclusion, BFR training may promote fat expenditure by increasing energy expenditure during exercise and EPOC.

In addition to the increased EPOC together with relevant fat oxidation, the redistribution of hydrocarbon source induced by high-physiological-load exercise facilitated the reduction in AVFA in the I group compared with the remaining groups [33,34]. As revealed from indirect evidence, active muscles after exercise possibly lead to transient elevation of competition against adipose tissues, such as abdominal fat depots, circulating postprandial hydrocarbons from different sources (fat, protein, and carbohydrate), so as to reconstruct tissues [35]. Additionally, the competition extent may depend on the physiological load in the process of exercise [33,34]. As a result, active muscles have a greater distribution of hydrocarbon-based nutrients after each meal, and this may achieve evident negative energy balance within abdominal fat cells, while promoting fat reduction [33,34].

Triglyceride hydrolysis to non-esterified fatty acids and glycerol represents the initial fat decomposition step (referred to as fat metabolism or lipolysis). Lipolysis plays a critical role since it represents the first step of oxidation, gluconeogenesis, and fat redistribution into skeletal muscle [34,36]. Similarly, fat reduction is suggested to be related to increased lipolytic hormone production due to the increased physiological load in the process of exercise, even though hormone-mediated lipolysis is not completely converted to the oxidation of fatty acids [37]. Our results showed that BFR increased the GH level after exercise, and the application of BFR in the interval phase could induce higher EPI levels. This supports prior studies that lipolytic hormones, mostly GHs and catecholamines, are elevated with the increase in physiological load during exercise [38]. It is further noted that the visceral fat area in the I group showed the most significant improvement, and the blood lactic acid level in the I group remarkably increased compared with the E and H groups, suggesting that visceral fat reduction is possibly achieved by stimulating EPI-induced lipolysis with high levels of blood lactic acid. As indicated by previous studies, the EPI-mediated phosphorylation of acetyl-CoA carboxylase (ACC) can reduce plasma triglyceride and abdominal visceral fat deposition via β-adrenergic receptors and activated protein kinase (AMPK) signaling pathways [39]. EPI activates AMPK in adipocytes and other tissues [40,41]. It induces the phosphorylation of ACC. ACC, one of the rate-limiting enzymes for fatty acid production, is conducive to reducing the accumulation of fat. Moreover, elevating plasma catecholamine contents in the process of exercise promotes lipolysis within adipocytes [40]. EPI, a catecholamine obtained from tyrosine, can be detected in the adrenal medulla and sympathetic nerve ends, which represent the endogenous ligands of adrenergic receptors. Generally speaking, hormone-sensitive lipase is characterized by its effect on lipolysis mediated by adrenergic receptors within white adipose tissues [42]. β-AR stimulation within brown adipose tissues promotes thermogenesis through the upregulation of uncoupling protein-1 [43]. Therefore, EPI can reduce the abdominal visceral fat mass through a variety of mechanisms.

Some limitations of this study should be noted. Firstly, the plasma volume was not corrected in hormone measurements, which could have interfered with the comparison of the increases in exercise-induced lipolysis hormone levels between timepoints and interventions. However, this interference was considered to be slight, because minor alterations of hematocrit and plasma albumin caused by exercise were observed in obese adults who participated in similar exercises and voluntarily hydrated. Secondly, for EPI and GH, the serum-lipolytic-hormone-release process was simply determined according to blood specimens harvested prior to, immediately after, and 30 min following exercise. Since the peak value of GH after exercise occurs at about 30 min, the possible different EPI responses at additional timepoints were not eliminated. In addition, the mechanism of BFR in improving visceral fat was not comprehensive, which should be further explored in future research.

## 5. Conclusions

Compared with HIIT alone, HIIT with BFR can better improve body-fat level and glucose metabolism. HIIT with BFR in the interval phase better reduces the abdominal visceral-fat level than in the exercise phase, and this may be due to the increase in lipolytic hormone level caused by the higher physiological load.

## Figures and Tables

**Table 1:** Physical characteristics of the participants and the changes in VO_2max_.

	C Group (*n* = 16)	H Group (*n* = 18)	E Group (*n* = 17)	I Group (*n* = 16)
Age (years)	21.5 ± 1.5	21.8 ± 1.8	22.1 ± 2.0	21.6 ± 1.9
Height (cm)	166.7 ± 9.0	168.0 ± 8.5	166.2 ± 8.9	166.6 ± 9.2
Weight (kg)	74.6 ± 7.5	74.4 ± 7.4	73.5 ± 9.3	75.4 ± 9.7
BMI (kg/m^2^)	26.8 ± 2.0	26.3 ± 1.1	26.5 ± 1.4	27.1 ± 1.5
VO_2max_ (mL/kg/min)				
0 weeks	31.3 ± 5.1	32.9 ± 5.1	32.0 ± 4.8	32.6 ± 6.1
4 weeks		34.7 ± 4.7 ^a^	34.2 ± 5.1 ^a^	34.9 ± 4.1 ^a^
8 weeks		36.1 ± 4.4 ^a,b^	36.0 ± 5.4 ^a,b^	37.1 ± 6.1 ^a,b^
12 weeks	31.7 ± 5.2	36.9 ± 5.9 ^a,b,d^	37.8 ± 5.3 ^a,b,c,d^	38.6 ± 6.2 ^a,b,c,d^

Note: ^a^ statistical difference with 0 weeks; ^b^ statistical difference with 4 weeks; ^c^ statistical difference with 8 weeks; ^d^ statistical difference with C group value.

**Table 2:** Calorie expenditure and intake.

	C Group		H Group		E Group		I Group	
	Pre	12 Weeks	Pre	12 Weeks	Pre	12 Weeks	Pre	12 Weeks
Estimated daily energy intake (kJ/day)	8236.9 ± 555.3	8288.9 ± 556.2	8113.9 ± 571.5	8066.4 ± 595.4	8159.0 ± 725.8	8220.0 ± 698.3	8123.6 ± 587.7	8162.1 ± 627.4
Estimated daily energy expenditure (kJ/day)	1952.9 ± 252.1	2002.8 ± 277.8	2016.4 ± 286.1	1929.6 ± 233.4	2024.1 ± 311.8	2033.5 ± 309.9	2004.2 ± 285.8	2041.4 ± 278.5

**Table 3:** Exercise intensity, HR, RPE, and LOP of each group.

	H Group			E Group			I Group		
	1–4 Weeks	4–8 Weeks	8–12 Weeks	1–4 Weeks	4–8 Weeks	8–12 Weeks	1–4 Weeks	4–8 Weeks	8–12 Weeks
Exercise intensity (W)	143.2 ± 31.5	159.4 ± 27.7 ^a^	175.4 ± 25.2 ^a,b^	146.8 ± 30.8	161.9 ± 27.1 ^a^	173.0 ± 26.9 ^a,b^	144.6 ± 32.5	167.4 ± 29.8 ^a^	177.1 ± 28.7 ^a,b^
HR (beats/min)	168.9 ± 12.5	171.1 ± 6.9	171.7 ± 12.1	173.8 ± 10.8	176.5 ± 9.1	174.1 ± 7.7	178.4 ± 11.0 ^c^	181.6 ± 9.2 ^c^	181.4 ± 13.4 ^c^
RPE	16.6 ± 0.9	16.5 ± 0.6	16.4 ± 0.9	16.5 ± 0.7	16.9 ± 0.7	16.6 ± 0.9	16.6 ± 1.1	16.9 ± 0.8	16.9 ± 0.9
LOP				204.8 ± 10.8	202.4 ± 10.3	203.8 ± 8.8	203.4 ± 13.3	208.8 ± 10.3	207.7 ± 13.6
40% LOP				81.9 ± 4.3	80.9 ± 4.1	81.5 ± 3.5	81.4 ± 5.3	83.5 ± 4.1	83.1 ± 5.4

Note: ^a^ significant difference compared with 1–6 weeks; ^b^ significant difference compared with 1–6 weeks; ^c^ significant difference compared with the H group; HR = heart rate; RPE = rating of perceived exertion; LOP = limb occlusive pressure.

**Table 4:** Changes of fat variables in each group.

	C Group		H Group		E Group		I Group		Two-Way ANOVA (Group, Time, Interaction)
	Pre	Post	Pre	Post	Pre	Post	Pre	Post	
% Body fat (%)	40.8 ± 3.5	41.0 ± 3.5	40.6 ± 3.6	38.4 ± 3.5 ^a^	40.2 ± 2.5	37.2 ± 2.4 ^a^	40.6 ± 2.6	37.5 ± 2.6 ^a^	(0.209, 0.001, 0.001)
	0.2 ± 0.6		−2.1 ± 0.9 ^b^		−3.0 ± 1.1 ^bc^		−3.1 ± 0.9 ^b,c^		
Whole-body FM (kg)	31.2 ± 5.7	31.3 ± 5.6	31.3 ± 4.4	28.8 ± 4.6 ^a^	30.9 ± 5.0	27.8 ± 4.8 ^a^	30.7 ± 3.8	27.3 ± 4.4 ^a^	(0.566, 0.001, 0.001)
	0.1 ± 0.6		−2.5 ± 1.1 ^b^		−3.1 ± 1.2 ^b^		−3.4 ± 1.2 ^b,c^		
Android FM (kg)	2.5 ± 0.5	2.5 ± 0.5	2.6 ± 0.6	2.4 ± 0.5 ^a^	2.5 ± 0.6	2.3 ± 0.5 ^a^	2.7 ± 0.6	2.4 ± 0.6 ^a^	(0.904, 0.001, 0.001)
	0.0 ± 0.1		−0.2 ± 0.1 ^b^		−0.2 ± 0.1 ^b^		−0.3 ± 0.1 ^b,c^		
Gynoid FM (kg)	5.7 ± 0.4	5.7 ± 0.5	5.9 ± 0.6	5.5 ± 0.5 ^a^	5.8 ± 0.6	5.4 ± 0.6 ^a^	6.0 ± 0.6	5.6 ± 0.6 ^a^	(0.875, 0.001, 0.001)
	0.0 ± 0.1		−0.4 ± 0.2 ^b^		−0.4 ± 0.1 ^b^		−0.4 ± 0.2 ^b^		
Trunk FM (kg)	16.6 ± 2.3	16.6 ± 2.2	16.9 ± 2.2	15.6 ± 2.0 ^a^	17.0 ± 1.8	15.1 ± 1.7 ^a^	17.5 ± 2.0	15.6 ± 2.1 ^a^	(0.867, 0.001, 0.001)
	0.0 ± 0.2		−1.3 ± 0.6 ^b^		−1.9 ± 0.4 ^b,c^		−1.9 ± 0.7 ^b,c^		

Note: ^a^ significant difference compared with Pre; ^b^ significant difference compared with C group; ^c^ significant difference compared with the H group; FM = fat mass.

## Data Availability

The data presented in this study are available on request from the corresponding author.
